# Effects of Cyclooxygenase Inhibitors in Combination with Taxol on Expression of Cyclin D1 and Ki-67 in a Xenograft Model of Ovarian Carcinoma

**DOI:** 10.3390/ijms13089741

**Published:** 2012-08-03

**Authors:** Wei Li, Jia-Hui Cai, Jun Zhang, Yun-Xian Tang, Liang Wan

**Affiliations:** Department of Gynecology, Nanjing Medical University of Hangzhou Hospital, 261 Huansha Road, Hangzhou 310006, Zhejiang, China; E-Mails: caijhgb@163.com (J.-H.C.); viola0116@163.com (J.Z.); tyxshuixian@163.com (Y.-X.T.); wanliang20069@126.com (L.W.)

**Keywords:** ovarian carcinoma, SC-560, celecoxib, taxol, cyclin D1, cell proliferation

## Abstract

The present study was designed to investigate the effects of cyclooxygenase (COX) inhibitors in combination with taxol on the expression of cyclin D1 and Ki-67 in human ovarian SKOV-3 carcinoma cells xenograft-bearing mice. The animals were treated with 100 mg/kg celecoxib (a COX-2 selective inhibitor) alone, 3 mg/kg SC-560 (a COX-1 selective inhibitor) alone by gavage twice a day, 20 mg/kg taxol alone by intraperitoneally (i.p.) once a week, or celecoxib/taxol, SC-560/celecoxib, SC-560/taxol or SC-560/celecoxib/taxol, for three weeks. To test the mechanism of the combination treatment, the index of cell proliferation and expression of cyclin D1 in tumor tissues were determined by immunohistochemistry. The mean tumor volume in the treated groups was significantly lower than control (*p* < 0.05), and in the three-drug combination group, tumor volume was reduced by 58.27% (*p* < 0.01); downregulated cell proliferation and cyclin D1 expression were statistically significant compared with those of the control group (both *p* < 0.01). This study suggests that the effects of COX selective inhibitors on the growth of tumors and decreased cell proliferation in a SKOV-3 cells mouse xenograft model were similar to taxol. The three-drug combination showing a better decreasing tendency in growth-inhibitory effect during the experiment may have been caused by suppressing cyclin D1 expression.

## 1. Introduction

Ovarian cancer represents the leading cause of death among gynecological malignances, and because of its insidious onset, most women with this disease present with advanced stage disease at the time of diagnosis. Although the quality of cytoreductive surgery, as well as development of novel drugs and new chemotherapy regimens for ovarian cancer therapy have been improved, long-term survival rates for patients with advanced epithelial ovarian carcinoma remain disappointing, and ongoing efforts have aimed to develop more effective primary therapy [1]. Since the early 1990s, taxol has been used to treat ovarian cancers [2]. Taxol belongs to a family of microtubule-targeting drugs called the taxanes [3], which work by promoting assembly and stabilization of microtubules preventing depolymerization.

However, tolerance to taxol in ovarian cancer cells has been observed [4]. Cyclooxygenase(COX)-2 overexpression has been found to be associated with chemotherapy resistance [5], and its overexpression might reduce the efficacy of taxol [6]. In addition, Subbaramaiah *et al.* also found that taxol could induce COX-2 mRNA expression and increase COX-2 protein levels in epithelial and tumor cell lines [6]. COX-2 overproduction induced by taxol may therefore cause undesirable effects. However, another study showed that overexpression of COX-2 predicts less susceptibility to platinum-based regimes but is not associated with response to platinum/paclitaxel [7]. COX-2 is one of the two isoforms of COX, which are the rate-limiting enzymes of the prostaglandins. It has been identified as being involved in the onset and progression of a variety of malignancies [8], including ovarian cancers [1]. Many studies found that selective COX-2 inhibitors could enhance the response to taxol in cancers [9], such as non-small-cell lung cancer [10] and ovarian cancer [11]. Another isoform of COX is COX-1, which is a constitutive form of the enzyme [12]. Gupta *et al.* [13] found that COX-1 was overexpressed in ovarian cancers *in vivo* and a later study showed its overexpression could be inhibited by COX-1 selective inhibitors in a mouse model of epithelial ovarian cancer [14]. These findings suggest that COX may play an important role in carcinogenesis and could be targeted for anti-tumor therapy. Nowadays, scholars have investigated the effects of COX inhibitors in combination with taxol on antiangiogenesis [9], apoptosis and proliferation [11]; however, the exact mechanism remains inconclusive.

Cyclin D1, a cell cycle protein, is a well-established human oncogene: A recent census concluded that there was substantial evidence for the involvement of cyclin D1 amplification and overexpression in cancers [15]. Moreover, in some studies the correlation between cyclin D1 expression and proliferation was echoed in carcinomas [16,17]. A recent study showed the deregulation of cyclin D1 expression could directly lead to some of the hallmarks of cancer by causing proliferation, and this could be a mechanism-based targeted therapy to treat human cancers [18]. In addition, it was previously reported that COX-1 [13], COX-2 [19] and cyclin D1 [20] were all up-regulated in ovarian cancer, and downregulation of cyclin D1 expression via a COX-2 dependent mechanism by celecoxib could be a potential mechanism to inhibit ovarian cancer growth [21]. Therefore, it is reasonable to believe that a decrease in cyclin D1 could be potentially effective in inhibiting proliferation of tumor cells. In this study, we hypothesized that the addition of COX inhibitors could enhance the antitumor effect of taxol on xenograft ovarian cancer by reducing the expression of cyclin D1 and decreasing cell proliferation.

## 2. Results and Discussion

### 2.1. Inhibition of Ovarian Cancer Growth

**Figure 1:**
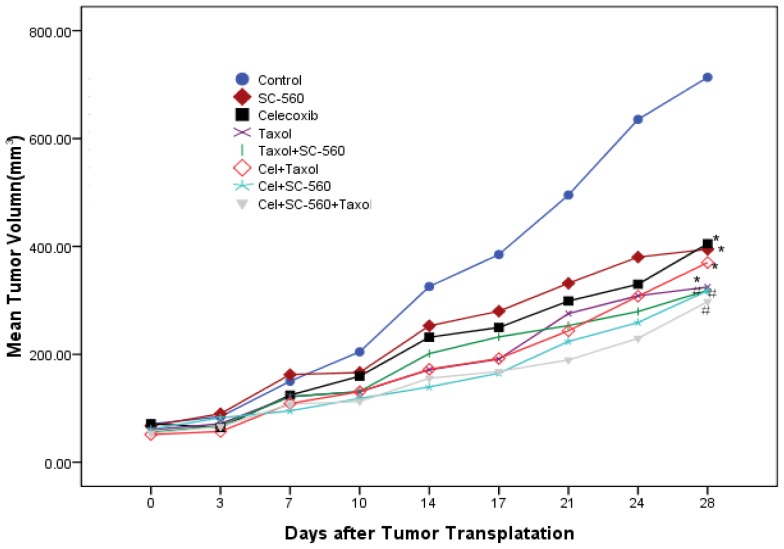
Effects of SC-560, celecoxib or/and taxol on tumor growth *in vivo*. The inhibitory of SC-560, celecoxib and taxol on tumor growth were determined in an ovarian cancer model using SKOV-3 cells. After 7 days to allow tumor establishment, mice were treated with SC-560, celecoxib and taxol. Treatment was continued for 21 days. The average tumor volume in all the drug-treated mice was significantly different from vehicle-treated mice at day 28. Statistical significance was determined using Least Significant Difference (LSD)-*t* test. * *p* < 0.05, **^#^** *p* < 0.01.

To test whether COX inhibitors or taxol could inhibit ovarian cancer growth, we used the human ovarian carcinoma cell line SKOV-3. The tumor growth in the control group increased throughout the period examined. Data in Figure 1 show the relative effect of SC-560, celecoxib or/and taxol treatment. At the end of the experiment, treatment with SC-560, celecoxib and taxol resulted in mean tumor volumes of 405.10 mm^3^, 394.75 mm^3^ and 324.79 mm^3^, respectively, while the mean tumor volume in control mice was 713.51 mm^3^; tumor growth was significantly reduced when treated with these drugs alone compared with the control group (*p* < 0.05). Under similar conditions, tumor volume in the three-drug combination group was reduced by 58.27% to 297.78 mm^3^ compared with control mice (*p* < 0.01). The inhibitory effect of the three-drug combination group showed a better decreasing tendency in growth-inhibitory effect compared with the independent group. No toxicity was observed in any of the animals, as measured by weight gain/loss as well as gross pathological examination of the gastrointestinal tract of the animals at necropsy.

### 2.2. Cyclin D1 Expression in Tumors

**Figure 2:**
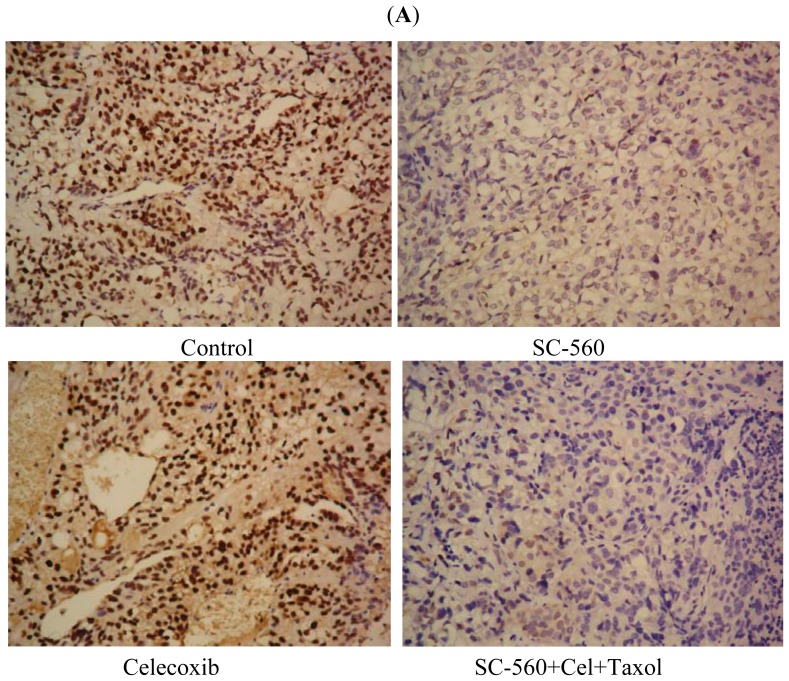

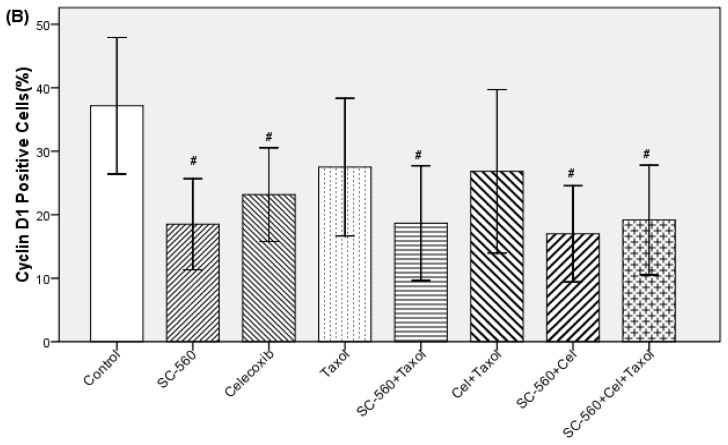
Effects of cyclooxygenase (COX) inhibitors and taxol on cyclin D1 activation in SKOV-3 xenograft tumors. (**A**) Representative pictures of cyclin D1 immunohistochemical staining of tumors. The expression of cyclin D1 in tumor sections was substantially lower when the mice were exposed to SC-560, celecoxib and the combination of the three drugs than the control. Magnification is ×400; (**B**) The results show that the positive rates of cyclin D1 in tumors of mice treated with SC-560 or/and celecoxib, and the combination of the three drugs were decreased. **^#^** *p* < 0.01 compared with control.

To evaluate the expression of cyclin D1 in SC-560, celecoxib or/and taxol-treated mice, protein changes in drug-treated xenograft tumors were detected by immunohistochemistry analysis. The expression of cyclin D1 in tumor sections was substantially lower when the mice were exposed to SC-560, celecoxib or the combination of the three drugs, compared with the control (Figure 2A). The expression positive rate of cyclin D1 was 37.17% ± 10.24% in the control, while 18.5% ± 6.83%, 23.17% ± 7.02%, 18.67% ± 8.62%, 17.00% ± 7.21% and 19.17% ± 8.23% of cells in the group treated with SC-560, celecoxib, SC-560/celecoxib, SC-560/taxol, or the combination of the three drugs, respectively; they all showed significantly decreased expression levels of cyclin D1 compared with the control (*p* < 0.01; Figure 2B). No variability among the drug-treated groups was observed.

### 2.3. SC-560, Celecoxib and Taxol Inhibit Tumor Cell Proliferation

**Figure 3:**
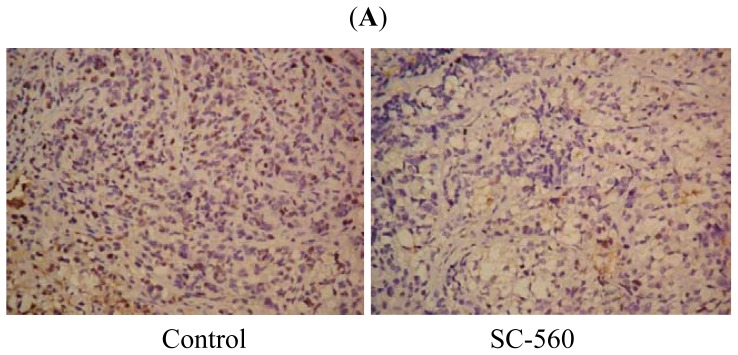

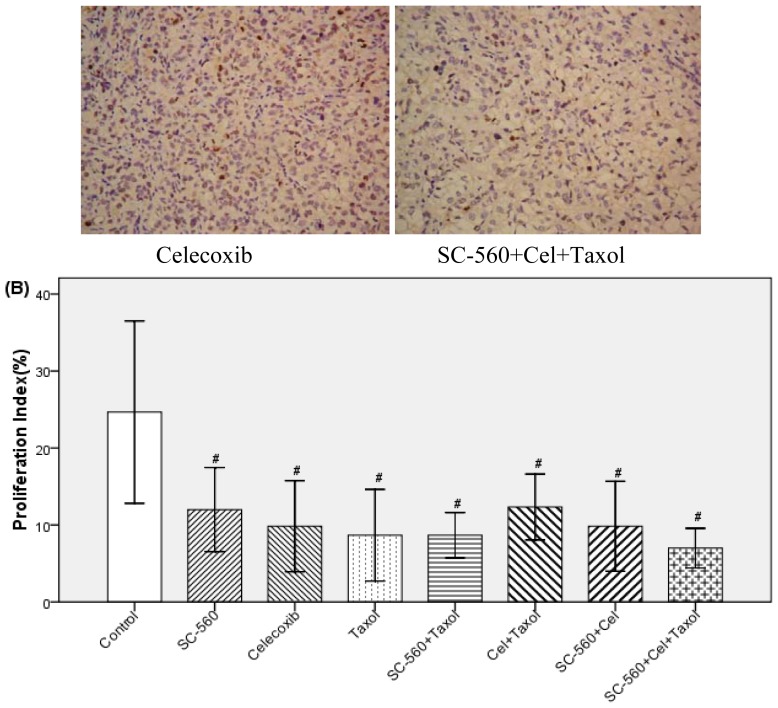
Cell proliferation in xenograft tumors of nude mice treated with SC-560, celecoxib or/and taxol. (**A**) Immunostaining of cell proliferation (Ki-67) by immunohistochemistry. The population of Ki-67-positive cells in tumor sections was substantially lower when the mice were exposed to SC-560, celecoxib and the combination of the three drugs than the control. Magnification is ×400; (**B**) The proliferation index was determined by the percent of Ki-67-positive cells. Proliferation index illustrates the proliferation of SC-560, celecoxib and taxol on tumors. **^#^** *p* < 0.01 compared with control.

We assessed cell division in allografted tumors treated with SC-560, celecoxib, taxol or the combination by proliferation-associated nuclear antigen (Ki-67) staining. The population of Ki-67-positive cells in tumor sections was substantially lower when the mice were exposed to SC-560, celecoxib and the combination of the three drugs than the control (Figure 3A). Data for the proliferation index of eight groups are shown in Figure 3B. In the SC-560, celecoxib or taxol groups, the proliferation index were 12.00% ± 5.22%, 9.83% ± 5.64% and 8.67% ± 5.68%, respectively while 24.67% ± 11.29% was observed in the control group; the independent groups or their combinations groups were all statistically significant compared with that of the control group (*p* < 0.01). No variability among the drug-treated groups was observed.

### 2.4. Correlation of Tumor Cell Proliferation with Cyclin D1 Expression

**Figure 4:**
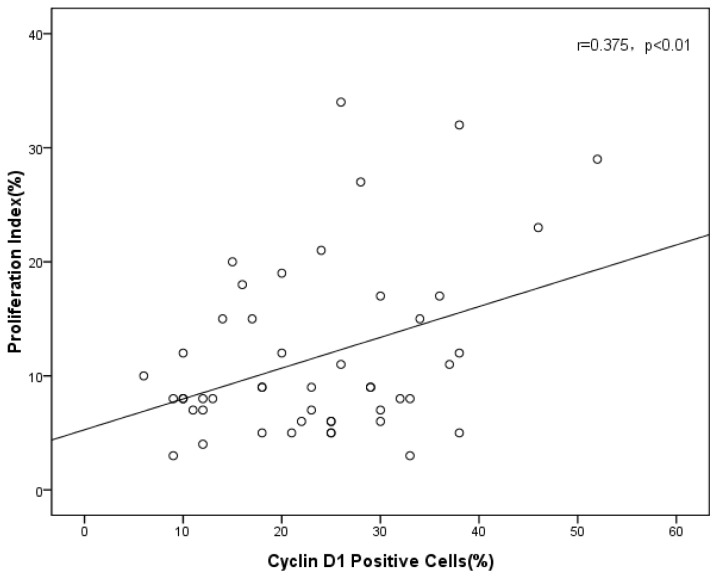
Correlation of tumor cell proliferation index with cyclin D1 positive rates (*r* = 0.375, *p* < 0.01).

We assayed tumor cell proliferation index and cyclin D1-positive rates in allograft tumors. The results shown in Figure 4 show a positive correlation (*r* = 0.375, *p* < 0.01) between proliferation and cyclin D1 expression in the tumors.

### 2.5. COX Expression

**Figure 5:**
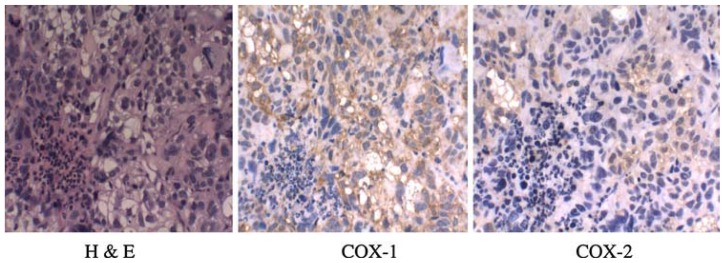
Immunohistochemical analysis of COX-1 and COX-2 expression in untreated tumor samples.

The untreated tumors were analyzed for expression of both COX isoforms. The results of immunohistochemical analysis show that the levels of COX-1 proteins were substantially higher than the COX-2 levels in these tumor samples (Figure 5).

### 2.6. Discussion

Treatment with novel drugs that selectively interfere with an important pathway controlling cancer cell proliferation, in combination with conventional anticancer treatments such as chemotherapy, has generated enormous clinical interest [22]. This study demonstrates that COX inhibitors in combination with taxol showed a better decreasing tendency in growth-inhibitory effect. This might be associated with reduced cyclin D1 expression.

Taxol is known as a front-line agent for ovarian cancer chemotherapy; however, long-term treatment often results in chemoresistance [4]. Studies have shown that involvement of COX-2 is associated with chemoresistance [5,23], and that taxol itself could induce COX-2 and prostaglandin biosynthesis, thus theoretically reducing its own cytotoxic effect. These findings suggest that COX-2 expression could play a role as an indicator of chemoresistance in ovarian cancer, and combination of a selective COX-2 inhibitor with taxol would overcome any decreases in efficacy related to the induction of COX-2 by taxanes [10]. Celecoxib has potent anti-tumor activity in a wide variety of human epithelial tumor types, and was most effective in decreasing tumor cell growth at the lowest dosages [24]. Furthermore, the combination of celecoxib and taxol has been used to treat carcinomas [10,25], and has previously been shown to be safe in a phase II study and has been tested with other agents with similarly good safety results [26]. SC-560 has been demonstrated to inhibit the overexpression of COX-1 in ovarian cancers [14]. However, studies on the combination of COX-1 selective inhibitor and taxol for cancer treatment have been rare. In our previous study [27], we found the combination of celecoxib and SC-560 lead to a greater efficacy than the individual agents administered alone. In this research, we also observed the three-drug combination showed a better decreasing tendency in growth-inhibitory effect. The result was better reflected in the effect of cyclin D1.

Induction of cyclin D1 is the rate-limiting step during the cell cycle, particularly in the transition from G1 to S phase. The G1/S checkpoint is frequently altered in many epithelial tumors and may confer growth advantage and enhanced tumorigenesis [28]. Cyclin D1 degradation is sufficient to induce G1 cell cycle arrest, and it might be a potential chemopreventive and chemotherapeutic target of ovarian cancer [29]. Substantial evidence for the involvement of cyclin D1 overexpression in cancers has been found [15], and research has demonstrated that an estimated 26% of sporadic epithelial ovarian cancers overexpress cyclin D1 [20]. Our previous trial presumed that celecoxib could inhibit the expression of cyclin D1 by a COX-2-dependent mechanism [21]. Moreover, not only celecoxib but also SC-560 could suppress the expression of cyclin D1 and inhibit proliferation in colon cancer cell lines [30]. Research has shown that diverse compounds, some with potential therapeutic application, lead to enhanced cyclin D1 degradation [31]. Therefore, cyclin D1 knockdown may provide a potential gene therapy approach [32]. In this research, we investigated whether treatment with SC-560/taxol and the combination of all three drugs significantly decreased the expression levels of cyclin D1. These findings suggested that the celecoxib- and SC-560-enhanced antitumor effect of taxol may in part be mediated through influencing the cell cycle. Shan J. *et al*. also showed promoting cyclin D1 degradation inhibited proliferation in cancer cells overexpressing cyclin D1 but not in normal fibroblasts, suggesting that targeting cyclin D1 degradation could be an effective, cancer-specific therapy [33].

Cyclin D1 has been proved to be associated with proliferation [16,17], and a recent study reported that cyclin D1 performs multiple functions as an oncoprotein through enhancement of proliferation and resistance to apoptosis, and may also contribute to chemoresistance in glioma [32]. High expression of Ki-67 has been found to indicate a poor prognosis in ovarian cancer [34], and it is positively correlated with tumor grade and may contribute to the identification of aggressive ovarian carcinomas [35]. A recent study reported that sustained proliferative signaling and activated invasion and metastasis are considered to be hallmarks of cancers [18]. In this study, we have shown that the tumor growth inhibition by celecoxib, SC-560 or taxol was accompanied by a decrease in proliferation index, and there was a positive correlation between proliferation and cyclin D1 expression in the tumors. The results suggested that the drugs may inhibit cell cycle progression through the G1-S transition in SKOV-3 cells by decreasing the expression of cyclin D1 as one of its potential antiproliferative mechanisms. The hypothesis to explain the relationship between the decrease of cyclin D1 and cell cycle arrest at G1 is that the loss of cyclin D1 results in recruitment of p21 to cyclin E2-cdk2 complexes, inhibiting cdk2 activity, which prevents pRb hyperphosphorylation, and that the E2F promoters remain repressed by the bound pRb complex, resulting in G1 arrest [29]. This antiproliferative effects could be attributed to the inhibition of cell survival signaling in ovarian cancer. Inhibition of cell proliferation is believed to be responsible for the chemo-preventative effects of COX inhibitors [14].

## 3. Materials and Methods

### 3.1. Human Ovarian Tumors in Nude Mice

The human ovarian carcinoma cell lines SKOV3 in our experiments was chosen for its ability to mimic the progression of ovarian carcinoma when injected into *in vivo* mouse models and it could be well used to observe the antitumor effect [11,36]. The SKOV-3 cells were purchased from China Type Culture Collection and grown in the recommended media under standard condition. SKOV-3 cells were implanted subcutaneously in the dorsal skin (2 × 10^6^ cells) of female athymic nude mice (nu/nu, 7–8 weeks old). When the tumors became visible (7 days after inoculation), the mice were randomly separated into eight groups (6 mice in each group): control, celecoxib, SC-560, taxol, celecoxib/taxol, SC-560/celecoxib, SC-560/taxol and SC-560/celecoxib/taxol.

### 3.2. Dose and Administration Time of Drugs

COX inhibitors, SC-560 (Sigma Chemical Co. St. Louis, MO, USA), celecoxib (Pfizer Co. Groton, CT, USA) were administered by gavage and taxol (Bristol Myers Squibb SRL, Italy) was given by intraperitoneal (i.p.) in a 0.5 mL suspension of 0.5% methylcellulose (Sigma Chemical Co. St. Louis, MO, USA) and 0.025% Tween 20 (Sigma Chemical Co.) at a dose of 3 mg/kg (SC-560), 100 mg/kg (celecoxib) twice a day, 20 mg/kg (taxol) once a week. The doses of COX inhibitors were chosen for their specificity in inhibiting COX isotypes [37]. In a control group, mice were treated with physiological saline under similar conditions. Drugs or vehicle were administered for a period of 21 days, beginning on the day one week after the tumors became palpable.

### 3.3. Measurement of Tumor Volume

The tumor dimensions were measured twice a week using a linear caliper, and tumor volume was calculated using the equation *v* (mm^3^) = *a* × *b*2/2, where *a* is the largest diameter and *b* is the smallest diameter [38]. The animals were weighed weekly throughout the experiment. On day 28, all of the mice were sacrificed, and tumor tissue samples were collected and then fixed in 10% phosphate-buffered formalin solution for immunohistology or stored at −80 °C until analyzed. The tumor tissue samples were snap-frozen in liquid nitrogen before their storage at −80 °C.

### 3.4. Immunohistochemistry for Ki-67 and Cyclin D1

Proliferation index was evaluated by staining for Ki-67. At the same time, cyclin D1 protein was also detected by immunohistochemistry. Tumors were fixed in 10% neutral buffered formalin for 24–48 h prior to being embedded in paraffin. After deparaffinization, the tissue sections were heated at 121 °C for 15 min in 10 mM TrisHCl with 1 mM EDTA (pH 9.0). Endogenous peroxidase was blocked with 3% hydrogen peroxide in methanol for 10 min at room temperature. The samples were incubated with Ki-67 antibody (clone MIB-5 (M7248)) or cyclin D1 antibody (Santa Cruz Biotechnology, USA) for 90 min at room temperature. Then, the sections were incubated in EnVision reagent for 40 min and DAB/H2O2 for 8–12 min at room temperature. Proliferation was assessed by counting the number of Ki-67 positively staining nuclei and total number of cancer cells at 400× magnification in five representative regions of the tumor. Results are expressed as the proportion of positively staining cells over the total number of cells. For evaluation of the cyclin D1 staining, the tissues were scored for the protein by assessing the site of positive staining in the nucleus or cytoplasm. The status of nuclear expression of cyclin D1 was assessed by determining the percentage of positive cells stained in five fields of each tissue section at 400× magnification.

### 3.5. Immunohistochemistry for COX-1 and COX-2 Expression

In brief, formalin-fixed paraffin-embedded tumor sections (6 μm) were subjected to immunostaining using COX-1 or COX-2 (Santa Cruz Biotechnology, USA), as described above. Sections were deparaffinized and hydrated by sequential immersion in xylene and grade alcohol solutions. The section was then incubated with 3% hydrogen peroxide in methanol solution for 34 min to block endogenous preoxidase activity. For antigen retrieval, slides were pressured in the pressure cooker for 2 × 10 min for or COX-1 or COX-2 at maximum power in 0.01 M citrate buffer (pH 6.0). Immunohistochemical staining was performed using the streptavidin–biotin method.

### 3.6. Statistical Analyses

Statistical analysis was performed with SPSS software (SPSS Standard version 17.0, SPSS). Statistical significance among control and drug-treated groups on tumor growth were determined by Least Significant Difference (LSD)-*t* test. We used a Tukey’s honest significance (Tukey HSD) test for evaluation of the inhibitory activity on tumor cell proliferation and cyclinD1 expression. All the experimental data were expressed as means values ± SE. Results were considered statistically significant when *p* value < 0.05.

## 4. Conclusions

This study suggests that the effects of COX selective inhibitors on the growth of tumors and decreased cell proliferation in a SKOV-3 cells mouse xenograft model were similar to taxol. The three-drug combination showed a better decreasing tendency in growth-inhibitory effect during the experiment, which may be explained by the suppression of cyclin D1 expression. However, intense research efforts are required to explore the effects on xenograft growth and additional possible mechanisms of the combinatorial strategy of COX-selective inhibitors and taxol in ovarian cancer therapies by other experiment models.
